# Evaluation of diastolic function in newly diagnosed hyperthyroid patients with preserved left ventricular systolic function

**DOI:** 10.1097/XCE.0000000000000238

**Published:** 2020-11-19

**Authors:** Maryam Shojaeifard, Zahra Davoudi, Azam Erfanifar, Hamed Fattahi Neisiani, Sajad Erami, Hooman Bakhshandeh, Khadije Mohammadi

**Affiliations:** a Department of adult Echocardiography, Echocardiography Research Center, Rajaie Cardiovascular Medical and Research Center, Iran University of Medical Sciences; b Department of endocrinology, Loghman Hakim Hospital, Shahid Beheshti University of Medical Sciences, Tehran; c Shahid Sadughi Hospital, Shahid Sadughi University of Medical Sciences, Yazd; d Rajaie Cardiovascular Medical and Research Center, Iran University of Medical Sciences, Tehran; e Cardiovascular Research Center, Kerman University of Medical Sciences, Kerman, Iran

**Keywords:** diastolic dysfunction, hyperthyroidism, preserved heart failure, stress echocardiography

## Abstract

**Background:**

Diastolic dysfunction has been reported as a cardiovascular effect of hyperthyroidism, and is also supposed to be a cause of heart failure manifestations in the patients with preserved ejection fraction (EF).

**Methods:**

For evaluation of diastolic function in hyperthyroid patients and also diastolic function during performing exercise stress echocardiography, we conducted this study on 25 newly diagnosed hyperthyroid patients compared to 26 healthy subjects as control group. Diastolic function of the patients at rest was assessed using these parameters as follows: left atrium volume index, tricuspid regurgitation (TR) velocity, mitral inflow early diastolic flow (E wave), tissue Doppler of mitral annular velocity (e′), E/e′ ratio, and isovolumic relaxation time (IVRT); and during exercise stress echocardiography, we judged diastolic response by measuring E/e′ ratio and TR velocity at the peak of stress.

**Results:**

The mean age of the patients was 39.64 ± 12.23 years old and 52% of them were female. History of exertional dyspnea was found in 64% of patients. All the patients had normal diastolic function at the time of resting and there was no significant difference in diastolic parameters between the two groups except for IVRT, which was lower in hyperthyroid patients. Interestingly, no patients developed diastolic dysfunction during exercise stress echocardiography.

**Conclusion:**

Our finding did not show diastolic dysfunction in hyperthyroid patients neither at rest nor during exercise echocardiography and did not support the hypothesis that diastolic dysfunction can be a cause of exertional intolerance and dyspnea in the patients with preserved EF.

## Introduction

Hyperthyroidism is defined as a clinical condition that resulted from abnormal high serum thyroid hormone levels [1]. Any increase in the level of thyroid hormone leads to change in many systems of human body including cardiovascular system because thyroid hormone acts through genomic and non-genomic mechanisms to regulate cardiac function [2]. Cardiomyocytes have thyroid hormone nuclear receptors that when bound alter gene transcription, can regulate intracellular calcium metabolism and myofilaments. Nongenomic effects on cardiomyocytes include altering ion channel permeability and then leading to tachycardia [3].

Hemodynamic effects of hyperthyroidism increased heart rate (HR), reduced peripheral vascular resistance, and increased preload, stroke volume, and consequently cardiac output [4]. Thus cardiovascular manifestation are common among the patients and hyperthyroid patients may clinically develop some symptoms like exertional dyspnea, palpitation, orthopnea, paroxysmal nocturnal dyspnea and peripheral edema [1,2].

More common cardiac manifestations of hyperthyroidism that was documented in various studies are systolic hypertension, left ventricular (LV) hypertrophy, increased LV mass, heart failure, and cardiac arrhythmia including atrial fibrillation [1,2,5,6]; however, evaluation of LV diastolic function in hyperthyroidism showed conflicting results in different studies [7–11]. Accordingly, in some of them, diastolic dysfunction was supposed as a cause of heart failure symptoms in the patients with preserved ejection fraction (EF) [7,12], while in others, the enhanced diastolic function was observed [10]. Considering that frequently, symptom of diastolic dysfunction only occurs during exercise, as LV filling pressure is normal at rest, but it is increased with exercise [13]. So, exercise stress echocardiography is a useful modality to detect diastolic capacity reserve by providing insight into cardiovascular hemodynamic. In this study, we tried to assess diastolic function in the hyperthyroid patients using the latest guideline of the American Society of Echocardiography (ASE) [14] and also evaluation of diastolic function in response to exercise during stress echocardiography to see whether it cause exertional dyspnea or not.

## Methods

This case-control study was conducted on 25 newly diagnosed and untreated hyperthyroid patients in comparison to 26 healthy. Also, age and sex were matched in subjects as control group in the Rajaie Heart center echo lab. Patients were referred to us for evaluation of cardiovascular status from outpatient clinic of Loghman Hakim Hospital Endocrinology Department. The recruitment phase was done from January 2019 to January 2020, and the study protocol was approved by local research ethics committee and informed consent was obtained from all participants. Patients who were diagnosed hyperthyroid based on clinical and laboratory data, had no history of hypertension, diabetes mellitus, cardiovascular disease, hyperlipidemia, anemia, pulmonary, and neuromuscular disease, and take no medication were entered to the study. Control subjects were chosen among the people with normal stress echocardiography result who came to our echo lab for checkup or non-anginal chest pain and had no past medical history with normal lab test for thyroid function, lipid profile, blood sugar, and hemoglobin.

At first, we assessed the patients by electrocardiography (ECG) and conventional echocardiography, and exclude those who had valvular heart disease, any structural heart disease, LV systolic dysfunction by means of LVEF of less than 55% by Simpson method, pulmonary hypertension [systolic pulmonary artery pressure more than 35 mmHg using tricuspid regurgitation (TR) velocity], and nonsinus rhythm. Three of 28 patients who were referred to our center were excluded from the study due to LV dysfunction, pulmonary hypertension, and atrial fibrillation plus LV dysfunction.

### Stress echocardiography

A complete two-dimensional and Doppler echocardiography was done by an experienced operator at the time of resting in left lateral decubitus position, using the same machine (affinity 70 Philips with 1–5 MHz transducer) in terms of the ASE recommendation. LV end-diastolic volume, end-systolic volume, and EF were calculated from apical two- and four-chamber views based on the modified Simpson method. Diastolic parameters including mitral inflow velocities (E and A waves), mitral annulus tissue Doppler velocities (septal and lateral e′), left atrium volume index (LAVI), and peak TR velocity were measured by averaging in three consecutive cardiac cycles to estimate diastolic function in terms of the 2016 ASE guideline [14]. Isovolumic relaxation time (IVRT) and myocardial performance index (MPI) were also measured using tissue Doppler method. Then, the patient and control groups underwent an exercise stress echocardiography on treadmill using Bruce protocol for evaluation of functional class, ischemia and arrhythmia according to their symptoms. Endpoints for exercise were chest pain, dyspnea, exhaustion, target HR of more than 90% adjusted by age, and significant ST-segment deviation. Blood pressure and ECG were recorded at any stage. Peak stress images including five standard echocardiographic views (parasternal long- and short-axis views, apical four-chamber, three-chamber, and two-chamber views) were obtained immediately after cessation of test during 1 minute for evaluation of ischemia and speckle tracking strain analysis. TR peak velocity was obtained within 1 minute from peak stress, and E and e′ velocities were measured at time of 60–90 ms from peak when E and A, e′ and a’ waves were not fused in HR about 120 beats per minute.

### Definition

Hyperthyroidism was diagnosed when serum-free thyroxine (T4) and triiodothyronine (T3) were more than upper limit of laboratory range, and thyroid-stimulating hormone (TSH) was less than 0.1 mIU/ml (T3 and T4 were measured by radioimmunoassay and TSH by immunometric method using commercially available kits).

Based on the ASE guideline, for evaluation of diastolic function in the patients with preserved EF we considered four criteria including: (1) septal e′ velocity <7 cm/s or lateral e′ <10 cm/s, (2) average E/e′ ratio >14, (3) LAVI >34 ml/m^2^, and (4) peak TR velocity >2.8 m/s. Diastolic function was normal when more than half of four variables were negative. LV diastolic dysfunction was present when more than 50% of indices were positive, and diastolic function was indeterminate if half of parameters were positive.

Stress echocardiography test was considered to be positive for diastolic dysfunction when all of following parameters were present in peak stress: (1) average E/e′ >14 or septal E/e′ >15, (2) peak TR velocity >2.8 m/s, and (3) septal e′ velocity <7 cm/s or lateral velocity <10 cm/s at baseline.

IVRT and MPI were calculated by tissue doppler method when myocardial velocities were recorded using spectral pulse doppler from mitral annular level in apical four chamber view. MPI was calculated as sum of IVRT and isovolumic contraction time was divided by ejection time.

### Statistical analysis

The results of quantitative variables with normal distribution were expressed as mean ± SD, and numerical variables without normal distribution were expressed as median with interquartile range. Qualitative variables were reported by number and percentage. To compare the numerical variables with and without normal distribution, ‘independent samples *t*-test’ and ‘Mann–Whitney *U* test’ were used, respectively. Also, chi-square test was used to compare nominal variables. All the tests were performed using IBM SPSS Statistics v. 22 at a 95% confidence level.

## Results

Twenty-five newly diagnosed hyperthyroid patients and 26 healthy subjects as control group were enrolled in this study. Mean age of the hyperthyroid patients was 39.64 ± 12.23 years old and 52 (*n* = 13) of them were females. The etiology of hyperthyroidism was Graves (80%), toxic multinodular goiter (12%), toxic adenoma (4%), and subacute thyroiditis (4%). Cardiovascular symptoms and signs include the following palpitation (44%), exertional dyspnea (64%), and atypical chest pain (20%). Baseline characteristics of the hyperthyroid patients are listed in Table 1.

**Table 1:** Baseline characteristics of the hyperthyroid patients

Characteristics	Value
Age, mean ± SD (years old)	39.64 ± 12.23
Female sex, *n* (%)	13 (52.0)
BSA (m^2^)	1.81 ± 0.20
TSH, mean (range)	0.068 (0.001–0.1)
Duration of symptoms(month), median (IQR)	5.5 (3.7–12)
Etiology of hyperthyroidism, *n* (%)	
Graves	20 (80.0)
Toxic multi nodular goiter	3 (12.0)
Toxic adenoma	1 (4.0)
Subacute hyper thyroiditis	1 (4.0)
Symptoms, *n* (%)	
Palpitation	11 (44.0)
Exertional dyspnea	16 (64.0)
Atypical chest pain	5 (20.0)

BSA, body surface area; IQR, interquartile range; TSH, thyroid-stimulating hormone.

In comparison between hyperthyroid and control groups, there was no significant difference in clinical parameters such as age, sex, and body surface area, and two groups were matched. Hemodynamic parameters assessment showed that the hyperthyroid patients had a higher HR compared to control group at rest, but both SBP and DBP were not significantly different between the two groups. All the subjects were in sinus rhythm without any significant ST-segment deviation.

In echocardiographic evaluation of diastolic function, we found that no patient had septal e′ <7 or lateral e′ <10 cm/s and mean values were 9.86 ± 2.1 and 13.51 ± 2.9, respectively. Mean E/e′ at rest in the hyperthyroid patients was 7.09 ± 1.1 that was in normal range and also was not elevated in none of the individuals. Also, TR velocity and LAVI met criteria for diastolic dysfunction in no patient; so, regarding the ASE guideline definition, no one had diastolic dysfunction at rest (Table 2).

**Table 2:** Comparison of clinical characteristic, hemodynamic, and echocardiographic parameters at rest between the hyperthyroid and control groups

	Hyperthyroid	Control	*P* value
Age (years old)	39.64 ± 12.23	40.54 ± 10.35	0.806
Sex, *n* (%) Female	13 (52.0%)	14 (53.8%)	0.895
Male	12 (48.0%)	12 (46.2%)	0.895
BSA (m^2^) HR (bpm)	1.81 ± 0.20 86.24 ± 7.81	1.77 ± 0.15 72.23 ± 10.13	0.436 0.000
SBP (mmHg)	125.00 ± 10.3	115.62 ± 23.7	0.09
DBP (mmHg)	77.72 ± 7.80	73.85 ± 9.30	0.12
Septal e′ at rest (cm/s)	9.86 ± 2.1	9.40 ± 2.0	0.565
Lateral e′ at rest (cm/s)	13.51 ± 2.9	13.17 ± 3.3	0.767
LAVI (ml/m^2^)	22.80 (21.00–25.15)	22.85 (20.75–24.85)	0.601
E/e′	7.09 ± 1.10	7.3 ± 1.69	0.613
TR velocity (m/s)	2.27 (1.97–2.61)	2.2 (1.97–2.3)	0.286
PAP (mmHg)	25.76 ± 5.74	23.9 ± 3.76	0.181
IVRT (ms)	63.4 ± 13.35	74.3 ± 12.01	0.008
MPI	0.52 ± 0.08	0.50 ± 0.09	0.487

HR, heart rate; IVRT, isovolumic relaxation time; LAVI, left atrium volume index; MPI, myocardial performance index; PAP, pulmonary artery pressure; TR, tricuspid regurgitation.

Diastolic indices including septal and lateral mitral annular velocities, TR velocity, E/e′ ratio, LAVI, MPI, and pulmonary artery pressure (PAP) showed no notable difference between the two groups; IVRT was the only parameter that was lower in the hyperthyroid patients compared to the control group (*P*-value = 0.008; Table 2).

During performing stress echocardiography, all subjects completed stress test in view of reaching at least 90% of maximum predicted HR, and no one had developed evidence of ischemia in echocardiography or ECG. About 17 patients (68%) reported dyspnea at peak stress that was not restricting for exercise and no one had typical chest pain and dizziness. No significant ST-T change and arrhythmia were induced.

For evaluation of diastolic function reserve, according to the mentioned criteria in previous section, no one had developed criteria of diastolic dysfunction in stress echocardiography and mean E/e′ ratio and TR velocity were 7.30 ± 1.37 and 2.6 (2.39–2.88), respectively. There was also no significant difference between the two groups (Table 3).

**Table 3:** Comparison of echocardiographic diastolic parameters at maximal exercise between the hyperthyroid and control groups

	Hyperthyroid	Control	*P* value
E/e′			
Maximal exercise	7.30 ± 1.37	7.07 ± 1.25	0.536
Δ E/e′	−0.20 ± 1.53	−0.22 ± 1.23	0.330
TR velocity (m/s)			
Maximal exercise	2.6 (2.39–2.88)	2.57 (2.46–2.70)	0.627
ΔTR velocity PAP (mmHg)	0.4 (0.33–0.55)	0.4 (0.3–0.57)	0.797
Maximal exercise	33.36 ± 7.66	31.77 ± 5.24	0.332
Δ PAP	7.60 ± 3.95	7.8 ± 3.21	0.808
IVRT (ms)			
Maximal exercise	46.80 ± 9.25	54.73 ± 12.69	0.013
Δ IVRT	−16.60 ± 13.16	−19.53 ± 12.05	0.563
MPI			
Maximal exercise	0.45 ± 0.08	0.45 ± 0.1	0.747
Δ MPI	−0.06 ± 0.09	−0.05 ± 0.08	0.831

IVRT, isovolumic relaxation time; MPI, myocardial performance index; PAP, pulmonary artery pressure; TR, tricuspid regurgitation.

## Discussion

Cardiovascular symptoms such as exertional dyspnea and impaired exercise tolerance are common in the hyperthyroid patients [1,15]. In our study, 64% of the patients had a history of exertional dyspnea. Probable explanations for exertional symptoms include impaired skeletal muscles function [16], ineffective oxygen utilization, respiratory muscle weakness and increased ventilator drive to breathe, increased airway resistance, diminished lung compliance, and heart failure [15]. In Raphael’s study [2], heart failure with the reduced EF was seen in 6% and heart failure with the preserved EF was seen in 10% of the patients. Siu CW *et al*. reported heart failure presentation in 5.8% of the hyperthyroid patients that only in 50% of them, there was reduced EF [17], and they proposed that diastolic dysfunction is the reason of HF symptoms in others.

We found that no hyperthyroid patient had diastolic dysfunction that was in contrast with many previous studies, because diastolic dysfunction specially impaired relaxation (grade 1) was frequently reported in many previous studies evaluated the hyperthyroid patients [1,2,4,7,18]. The most important explanation for this discrepancy was that we used newer guideline criteria for defining diastolic dysfunction compared to previous studies that were based on the conventional indices, and also we excluded the patients with comorbidities that may affect diastolic function such as those with diabetes, hypertension, and coronary artery disease. We included newly diagnosed hyperthyroid patients who received no medication and this point was another difference between our study and previous studies that were done on the patients consuming anti-thyroid medications, and suggested that, maybe initiation of drugs induced diastolic change that need to be assessed in future studies.

By comparing diastolic parameters between the two groups, IVRT was the only index that significantly differed and other indices including E/e′, LAVI, TR velocity, septal, and lateral e′ were comparable. Enhanced diastolic function was also reported in Mintz G’s study [10], and their results about IVRT were similar to us, however, their study was done only at rest. Shorter IVRT in hyperthyroidism is due to lusitropic effect of thyroxin [19] through positively regulated sarcoplasmic Ca-ATPase because reuptake of calcium into the sarcoplasmic reticulum early in diastole can determine the rate of the left ventricle relaxation (IVRT).

In our study, diastolic function reserve was also assessed by exercise stress echocardiography using ASE guideline criteria. Accordingly, this evaluation was not performed in previous studies, and interestingly, we found that no one in hyperthyroid group had developed diastolic dysfunction criteria despite of reporting dyspnea by 68% of patients during exercise.

### Conclusion

Our finding did not show diastolic dysfunction in hyperthyroid patients neither at rest nor during exercise echocardiography and did not support the hypothesis that diastolic dysfunction can be a cause of exertional intolerance and dyspnea in the patients with preserved EF and normal PAP.

## Limitations

First, by considering that we excluded the patients with any comorbidity and those taken medications, the main limitation of our study was the small sample size. Second, we selected our cases from outpatient clinic and did not include more severe cases who may need hospitalization and probably with more severe cardiac involvement including diastolic dysfunction.

## Acknowledgements

**Conflicts of interest**

There are no conflicts of interest.
